# Scientific Items

**Published:** 1888-10

**Authors:** 


### SCIENTIFIC ITEMS.

**American Association for the Advancement of Science.**—This Society has embraced in its membership all or nearly all of the scientific men of America, and in the list of its officers—presidents, vice-presidents, and secretaries—the names of many of the illustrious scientific men of the country are enrolled.

To enumerate in systematic order the fields of research occupied by the various members of this association would be to formulate a classification of the sciences. "Atoms, mountains, and worlds, with all inorganic bodies and all inorganic motions, are to be examined in the study of the inorganic realm. The phenomena of nature are qualitative and quantitative, and out of quantitative relation the abstract science of mathematics has been developed, and this science of measurement is rapidly being applied to the qualitative sciences. There are many men engaged in mathematical researches. There are members who study the stars, compute their distances, and determine their courses, and who are seeking to solve the mysteries of the constitution of the bright sun and the pale moon; for on the chariot of light they drive through the storms of the greater orb and explore the dim fields of the lesser. Others with patient labor seek to discover the nature of light, of electricity, and of gravity—the mysterious force that impels the universe. There are others, many others, who are investigating the minute constitution of matter and determining its many forms. These forms are forever changing. The crystals of the rocks that make up the mountain mass are dissolved and their atoms redistributed in new forms, and chemical changes are in progress wherever human investigation can penetrate. There are others who are studying the surface of the earth—the lands and seas in all their places and forms. At the far North there is a region walled by ice, a million and a half square miles in extent; but even into this land of ice they penetrate. About the South Pole there is an area of seven million square miles inclosed by a barrier of ice—an unknown region into which the modern scholar is bound to enter."—*Scientific American.*

**Ants and their Ways.**—Much has been written about the industry and sagacity of the ant. Dr. Bonavia, in a recent number of the *Gardeners' Chronicle*, relates his observations on the habits of ants in India, in which he says:

"On one occasion I killed a wasp—the small yellow wasp that is so common in India. Soon after, I observed an ant moving about on the sill of a window. It struck me as a good opportunity to observe what steps this ant would take if brought in contact with the dead wasp. I placed the wasp in the path of the ant and watched the result. The ant, on finding the wasp, climbed over it and explored it thoroughly in all directions. Having satisfied itself that this was a good find, it descended, and ran down the wall of the window and across a very rough grass mat made of the Saccharum moonja (the moonj or surput matting of India). It galloped across the room over this rough surface as hard as it could go in a particular direction, disappeared under the wooden sill of a door. I still watched to see what would happen. Presently a long string of ants in single file came out and followed the exact course that the foraging ant had taken. They crossed the mat in the same course, and went up the wall straight to the wasp. I left them in peace, and some time after I found only the shell of the wasp. It is evident that single ants leave the nest, as scouts or explorers, on foraging expeditions, and go to long distances. By some scent left on their course, they are able to retrace their steps to their nest The ants in the nest, probably by some scent of the body found which the exploring ant brings with it, are made to understand that something good to eat has been found. Guided by the exploring ant, or by the scent it may have left in its track, the whole nest or a portion of it sallies out, and goes straight to the find.

**Ostriches.**—A correspondent at Cape Colony, South Africa, writes us as follows: A curious habit of these birds was witnessed on the farm Guilford, in the Queenstown district by the proprietor and some of his family and servants during the late rains.

The nest, which is merely a large flat, saucer-like hole in the ground, became flooded; and when the water did not directly drain off, the two parent birds began to drink it up till the nest was drained dry.

The poor hen bird was so full that she seemed quite sick; the cock, however, drank his full share as in duty bound, being the most asciduous in all matters pertaining to the incubator, always sitting on the eggs himself by night.—*S. American.*

**Snake Bite and Yellow Fever.**—Dr. Urias da Silveira has sent to the Medico-Chirurgical Society of Rio de Janeiro a quantity of vegatable substance which is very common in the provinces—Minas geraes and Barra mansa—and which, he says, he has used with great advantage in the bites of cobras, especially during the period in which the most serious symptoms—hemorrhages and ataxo-adynamic phenomena—appeared. He points out analogies between the effects of snake bite and of yellow fever, both of a symptomatic and pathological nature, and suggests that the drug he sent should be tried in cases of yellow fever.—*Ib.*

**Fire and Water-Proof Paper.**—A paper that resists the action of both fire and water has, it is said, been recently invented in Germany by a Herr Ladewigg. The manufacture is accomplished by mixing twenty-five parts of asbestos fiber with from twenty-five to thirty parts of aluminum sulphate, and the mixture is moistened with chloride of zinc and thoroughly washed in water. It is then treated with a solution of 1 part of resin soap in eight to ten parts of a solution of pure aluminum sulphate, after which it is manufactured into paper like ordinary pulp.—*Scientific American.*

